# The Role of the Histone Variant H2A.Z in Metazoan Development

**DOI:** 10.3390/jdb10030028

**Published:** 2022-07-01

**Authors:** Yasmin Dijkwel, David J. Tremethick

**Affiliations:** Transcription and Chromatin Group, Genome Sciences and Cancer Division, The John Curtin School of Medical Research, The Australian National University, Canberra 2601, Australia

**Keywords:** chromatin, chromatin remodeling, development, differentiation, epigenetics, H2A.Z, histone variant

## Abstract

During the emergence and radiation of complex multicellular eukaryotes from unicellular ancestors, transcriptional systems evolved by becoming more complex to provide the basis for this morphological diversity. The way eukaryotic genomes are packaged into a highly complex structure, known as chromatin, underpins this evolution of transcriptional regulation. Chromatin structure is controlled by a variety of different epigenetic mechanisms, including the major mechanism for altering the biochemical makeup of the nucleosome by replacing core histones with their variant forms. The histone H2A variant H2A.Z is particularly important in early metazoan development because, without it, embryos cease to develop and die. However, H2A.Z is also required for many differentiation steps beyond the stage that H2A.Z-knockout embryos die. H2A.Z can facilitate the activation and repression of genes that are important for pluripotency and differentiation, and acts through a variety of different molecular mechanisms that depend upon its modification status, its interaction with histone and nonhistone partners, and where it is deposited within the genome. In this review, we discuss the current knowledge about the different mechanisms by which H2A.Z regulates chromatin function at various developmental stages and the chromatin remodeling complexes that determine when and where H2A.Z is deposited.

## 1. Introduction

Multicellular eukaryotes comprise hundreds of different cell types, each performing a different specialized function. Because of the multitude of cell types, each with the same genome, the coordinated regulation of gene expression required to bring about differentiation from a single fertilized egg cell and then to maintain homeostasis for the life of the organism is necessarily complex. DNA is packaged into chromatin, which is ultimately linked to the establishment of a unique gene expression program for each cell type during development and differentiation [1]. Histones form the core structure of chromatin, called the nucleosome, in which DNA is wrapped around a histone octamer comprising an H3–H4 tetramer flanked by two H2A–H2B dimers [2]. Arrays of nucleosomes fold and compact into increasingly more complex higher-order structures that ultimately form chromosomes [3]. Linker histone H1 binds to the DNA entry and exit site of a nucleosome and with linker DNA to promote intra-nucleosome interactions and chromatin compaction [4]. Chromatin is a remarkable structure because, on the one hand, it enables over 2 m of DNA code to be highly compressed into a nucleus about 10 μm in diameter, while, on the other hand, it enables specific regions of the genome to become accessible in a spatial and temporal manner to allow the expression of distinct sets of genes needed for the formation of a multicellular organism [5].

Different epigenetic-based mechanisms regulate the function of chromatin. One mechanism includes histone posttranslational modifications (PTMs) [6], although many PTMs are the result of transcription. Another important mechanism is the substitution of core histones with their variant forms by histone chaperones and chromatin remodeling complexes [7]. These mechanisms control the structure of chromatin directly or indirectly through the recruitment of effector complexes, such as chromatin remodelers, and, therefore, determine whether a gene is assembled into an open and poised, or active, configuration (euchromatin) or a compacted and inactive structure (heterochromatin) [5].

H2A.Z is crucial for maintaining genomic integrity, regulating transcription, facilitating DNA double-strand break repair, and cell cycle progression, and is one of the most extensively studied histone variants [8,9,10,11]. The precise mechanism by which H2A.Z functions in these different cellular processes remains poorly understood, and the current understanding of this mechanism is complex and incomplete because H2A.Z acts through a variety of different molecular mechanisms in a context-specific manner.

H2A.Z has ~60% sequence identity with H2A and is highly conserved between organisms ranging from budding yeast to humans [10,12]. Notably, however, there are significant amino acid residue differences between budding yeast and metazoan H2A.Z, and a single histidine amino acid residue difference located on the surface of the nucleosome has been shown to be required for early *Xenopus laevis* development [13]. The main differences between H2A.Z and canonical H2A are in the L1 loop, which is responsible for stabilizing H2A–H2A interactions [14], and the docking domain and part of the C-terminal tail, which are responsible for the interaction with the H3–H4 tetramer needed to stabilize the histone octamer. Importantly, H2A.Z has an extended acidic patch [15] that promotes chromatin compaction [16,17]. The acidic patch of H2A.Z is essential for its function during early metazoan development [13,18].

There are two major paralogs of H2A.Z, H2A.Z.1 and H2A.Z.2, which are encoded by different genes. These histones differ by only three amino acid residues. One difference is in the L1 loop region (S38 in H2A.Z.1 and T in H2A.Z.2), which is thought to cause differences in their exchange rates in chromatin in cells [19]. Despite these minimal differences, H2A.Z.1 and H2A.Z.2 have been shown to play different roles in development, as described below.

**Figure 1:**
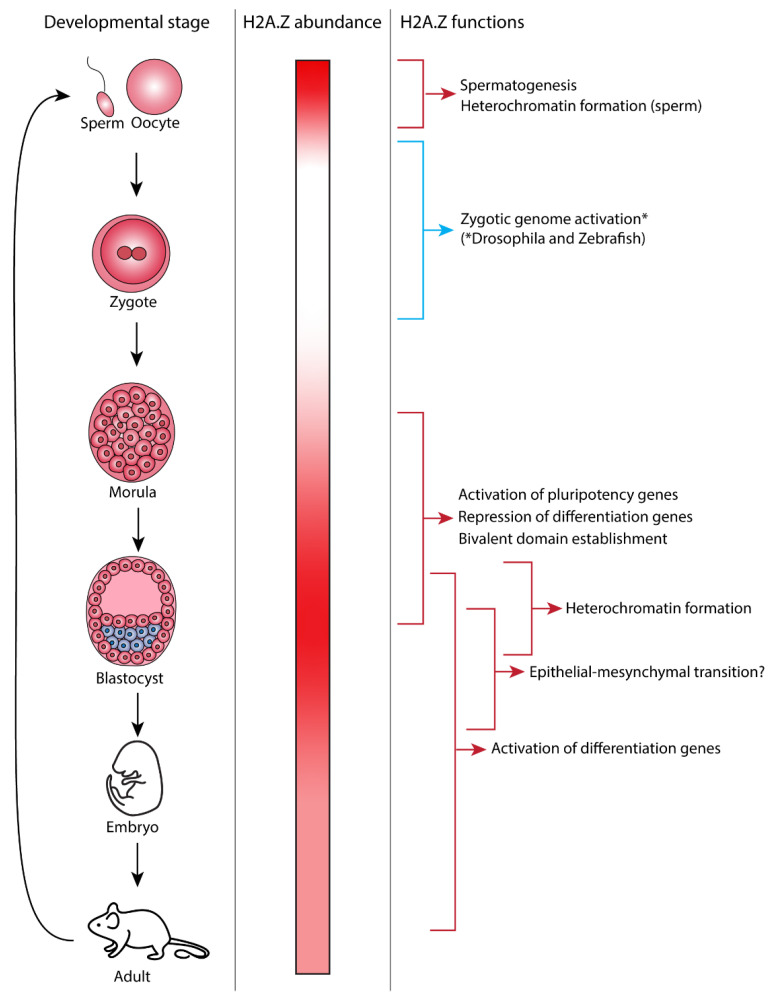
Developmental-stage-specific functions of H2A.Z. The different stages of mouse development are shown in the left panel. The middle panel shows the relative abundance of H2A.Z protein in each developmental stage. Maternal H2A.Z is present in the gamete and becomes depleted until H2A.Z becomes highly expressed in the blastocyst stage. Differentiation is associated with a decrease in H2A.Z abundance. The right panel shows the developmental processes that H2A.Z has been found to play a role in and depicts when during mouse development these processes occur. * Note that while this figure depicts mouse development, a direct role of H2A.Z in zygotic genome activation has only been shown in *Drosophila* and zebrafish to date [41,42].

Studies using different model organisms to examine the role of H2A.Z in development have found that this histone is expressed throughout the developmental process and is involved in specific developmental steps (Figure 1). Depletion of H2A.Z causes major defects at several stages of the differentiation process, although these defects differ between model organisms. H2A.Z-knockout mice embryos develop normally to the blastocyst stage and up to 5.5 days post coitum (dpc) but fail after implantation [20]. During this period, there is a high rate of proliferation associated with major differentiation events and the reorganization of the inner cell mass (ICM) followed by gastrulation. By 7.5 dpc, mutant embryos cannot be identified. H2A.Z is present in both the ICM and trophectoderm in 3.5 dpc embryos. In vitro, the ICM of 3.5 dpc H2A.Z-knockout embryos fails to differentiate. These findings suggest that H2A.Z plays a critical role following blastocyst formation [20]. In addition, H2A.Z has been found to regulate the differentiation of a number of different lineages, including the endoderm [21], neuronal cells and the brain [22,23,24,25], muscle [26,27,28], melanocytes [29], and intestinal cells [30,31]. Therefore, H2A.Z has multiple roles during development and differentiation (Figure 1).

Homologs of H2A.Z are also required for the development of other organisms. The *Caenorhabditis elegans* homolog of H2A.Z has been shown to play an essential role in development. H2A.Z is expressed in pluripotent cells [32] and in every cell of the developing *Caenorhabditis elegans* embryo [33]. The loss of H2A.Z causes embryonic lethality at the stage when maternal H2A.Z is depleted [33]. H2A.Z is essential for *Drosophila* development [34] and for normal larval fly hemocyte differentiation [35]. H2A.Z is also essential for sea urchin embryogenesis [32,36] and zebrafish development [37].

The fundamental unanswered question relates to the molecular mechanisms that underpin the requirement for H2A.Z during development and differentiation. The key features of H2A.Z that impact gene expression include its ability to alter the structure and stability of chromatin and the nucleosome, to change the surface of the nucleosome to recruit different types of reader proteins [8], and to provide an obstacle to elongation of the RNA pol II complex [38,39,40]. The ability of H2A.Z to influence patterns of gene expression relates to its ability to be deposited onto, or removed from, specific regions of the genome through the actions of histone chaperones and chromatin remodelers, most notably, at the transcription start site (TSS) and enhancers. The functions of H2A.Z can be expanded further by PTMs, crosstalk with other histone PTMs, or the histone variant H3.3 in the same or surrounding nucleosomes, whether H2A.Z is present as a single or double copy in the nucleosome (i.e., homotypic or heterotypic), and by the presence of different allelic isoforms or splice variants. The currently known mechanisms whereby H2A.Z regulates gene expression in development are described in detail below.

## 2. H2A.Z and Spermatogenesis

Gametogenesis is the process by which sperm or oocytes are produced during spermatogenesis and oogenesis, respectively. H2A.Z expression is developmentally regulated during spermatogenesis in mice and its expression increases at pachytene, when recombination and meiotic sex chromosome inactivation occur. Its expression peaks upon the completion of meiosis at the round spermatid stage, the stage with the highest level of transcription and when sex chromosome inactivation is maintained [43,44]. Although its exact role has not been studied extensively, the appearance of H2A.Z at the pachytene stage coincides with the major nuclear reorganization of the heterochromatic marks HP1α and H3K9me3, and their enrichment at the constitutive heterochromatin-containing chromocenters and the inactive XY body [43,45]. Notably, H2A.Z replaces another histone, the H2A variant macroH2A, on the X and Y chromosomes, to assemble facultative heterochromatin, indicating that H2A.Z plays a role in maintaining the sex chromosomes in a silenced state [43]. Therefore, during spermatogenesis, it appears that H2A.Z is involved in complex mechanisms that maintain and regulate heterochromatin structure and function.

## 3. H2A.Z and Zygotic Genome Activation

After fertilization, the maternal and paternal genomes unite. The resultant zygotic genome is quiescent, and it is the maternally provided mRNAs and proteins stockpiled in the oocyte that initiate development and allow the zygote to be programmed to a totipotent state. This is followed by a maternal-to-zygotic transition, whereby the genome is activated in a process called zygotic genome activation (ZGA). ZGA occurs in two transcriptional waves: a minor wave, in which a small number of genes are activated, and a major wave, in which many more genes are activated. These waves occur at different stages in different organisms. In humans, the minor wave occurs at the four-cell stage and the major wave at the eight-cell stage. By contrast, in mice, these waves occur at the one-cell and two-cell stages, respectively. In faster-growing animals, such as *Xenopus* and *Drosophila*, ZGA occurs earlier, within the first few hours after fertilization, reflecting the faster rate of cell division in faster growing animals.

During ZGA, chromatin is reprogrammed via different epigenetic-based mechanisms. In *Drosophila* and zebrafish, H2A.Z has been shown to be directly involved (Figure 1). In *Drosophila*, H2A.Z is deposited at a subset of promoters before the induction of ZGA and recruitment of RNA pol II [41]. Experimental depletion of chromatin-bound H2A.Z leads to a reduction in RNA pol II occupancy and transcription of H2A.Z-bound genes, and defects in ZGA. H2A.Z-positive genes are enriched for housekeeping genes, which suggests that H2A.Z is important for the activation of genes that control basic cellular processes during ZGA [41]. In zebrafish, H2A.Z is thought to prevent DNA methylation, which represses transcription, and to protect the genome from incorrect methylation [37,42,46]. ‘Placeholder’ nucleosomes are established that prevent DNA methylation temporarily but direct these sites for future methylation. These placeholder nucleosomes have been found to contain H2A.Z and H3K4me1 [42]. Placeholder nucleosomes can transition into two different states, repressed or bivalent. Bivalent domains are in a ‘poised’ state—neither in a permanent ‘on’ nor ‘off’ state—but become activated or repressed upon differentiation. Bivalent promoters often control genes that promote differentiation [47]. Misregulation of H2A.Z localization results in inappropriate gene transcription. In zebrafish, an increase in the amount of chromatin-bound H2A.Z causes gene activation, whereas a decrease causes gene repression in developmental genes [42].

On the other hand, in mice, H2A.Z has been found to be expressed at a low level during ZGA, but its expression increases during later stages of development [48,49,50]. Acetylated H2A.Z, a mark that correlates strongly with transcription, is not present during the murine two-cell stage [50]. H2A.Z-knockout mice develop normally until the blastocyst stage [20], which suggests that this histone may not play a major role in ZGA or, alternatively, that there is sufficient maternal H2A.Z. However, expression of a mutant form of H2A.Z, whose incorporation into chromatin is forced by the addition of the H2A.X C-terminal tail, causes a failure to extend to the blastocyst stage [48]. This suggests that the absence of H2A.Z may be necessary for blastocyst formation. Whether the chromatin remodelers that deposit H2A.Z (see below) are absent or not functional during ZGA is an interesting question to address in the future. Taken together, the findings discussed above indicate the importance of H2A.Z in ZGA and that the role of H2A.Z in early development is species dependent.

## 4. The Exit of Pluripotency and the Role of H2A.Z

The blastocyst comprises the pluripotent ICM and trophoblast stem cells that form the extraembryonic placenta. The ICM differentiates into the pluripotent epiblast and primitive endoderm before implantation in the uterus. After implantation, the ICM undergoes a wave of epigenetic reprogramming and associated changes in gene expression, necessary for the formation of the different cell lineages of the embryo. These changes include the coordinated repression of pluripotent genes and activation of differentiation and lineage-specifying genes. Mouse embryonic stem cells (mESCs), which are derived from the epiblast, have been used as an in vitro model system to study the roles of H2A.Z in regulating transcription before and after differentiation.

**Figure 2:**
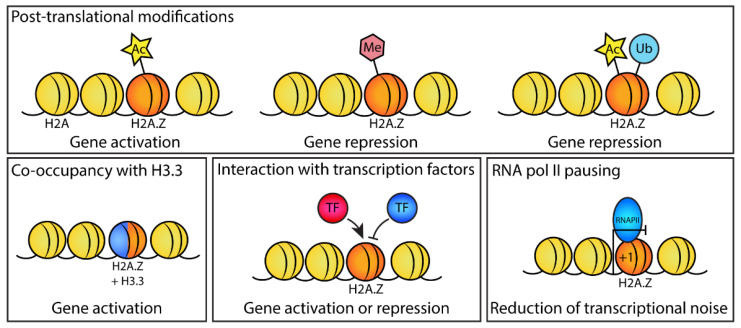
Mechanisms of H2A.Z in regulating gene expression during development. Different mechanisms by which H2A.Z affects gene expression during development, which include H2A.Z posttranslational modifications (acetylation, methylation, and ubiquitylation) that can promote or repress gene expression; the co-occupancy of H2A.Z with the histone variant H3.3, which in some cases can promote gene expression; the interaction of H2A.Z with stage-specific transcription factors (e.g., Nanog, CDX2, BRD2) to promote or repress their recruitment to chromatin; and inhibiting or dampening the transcription of active genes when H2A.Z is present at the +1 promoter nucleosome (the nucleosome directly downstream of the TSS) through promoting RNA pol II pausing.

The level of H2A.Z decreases upon differentiation in mESCs, which indicates that H2A.Z is important for maintaining the pluripotent state. Similarly, depletion of H2A.Z induces differentiation that is related to a reduction in pluripotency gene expression and the resultant activation of differentiation-specific genes [30,51,52,53,54]. However, as described below, H2A.Z is also required for the proper differentiation of specific cell types (Figure 1). Whether the loss of H2A.Z is a general feature of differentiation remains to be determined. H2A.Z acts through several mechanisms to either repress or facilitate gene expression in pluripotent cells and during the exit of pluripotency (Figure 2).

### 4.1. H2A.Z Acetylation Affects the Expression of Pluripotency Genes

H2A.Z is acetylated on its N-terminal tail at lysine residues K4, K7, K11, K13, and K15. It can be mono- or multi-acetylated [55,56] and acetylation at different residues has been shown to have the same effect [57], suggesting that charge rather than recognition by specific proteins is important for its function. Active genes have a higher proportion of acetylated H2A.Z (H2A.Zac) but generally contain less total H2A.Z than inactive genes [58,59]. In mice, H2A.Zac first appears at the four-cell stage after ZGA, increases further in the morula stage, and then decreases slightly in the blastocyst compared with the morula [50]. This pattern suggests that H2A.Zac positively regulates genes that are required for pluripotency during blastocyst development (Figure 1). Similarly, in mESCs, H2A.Zac is associated with active genes. H2A.Zac colocalizes with the active mark H3K4me3 at promoters and enhancers. Specifically, the expression of the pluripotency genes *Klf4*, *Tbx3*, and *Sox2* is dependent on the presence of H2A.Z. H2A.Z is also required for the recruitment of OCT4 protein to pluripotency genes to facilitate gene activation [52]. Whether preventing H2A.Z acetylation inhibits the expression of pluripotent gene expression has not been investigated.

### 4.2. H2A.Z Associates with Nanog to Promote Pluripotency

Another mechanism by which H2A.Z regulates transcription is through its association with transcription factors (Figure 2). For example, H2A.Z binds directly to Nanog [60], a key transcription factor responsible for maintaining mESC pluripotency. Nanog acts by downregulating genes that promote differentiation and upregulating genes that maintain pluripotency, in cooperation with transcription factors OCT4 and SOX2 [61]. The direct association of H2A.Z with Nanog stabilizes the Nanog protein and is thought to occur through inhibition of the ubiquitin–proteasome pathway. Together, this indicates that H2A.Z promotes pluripotency by also regulating Nanog protein stability [60]. In other systems, such as cancer cell lines, H2A.Z nucleosomes are also specifically bound by other interactors. These include, for example, the chromatin remodeling proteins PWWP2A [62], which is critical for neural crest differentiation and migration in Xenopus development [63] and BRD2, which binds preferentially to chromatin marked by acetylated histones H2A.Z and H4 and mediates transcriptional activation of androgen-responsive-regulated genes [64]. Although it is possible that these mechanisms also act in development, this has yet to be confirmed experimentally.

### 4.3. H2A.Z and H3.3 Coactivate Genes

The incorporation of H2A.Z and H3.3 into the same nucleosome has been shown to destabilize chromatin structure [65]. In mESCs, strong overlap of H2A.Z with H3.3 occurs at active promoters and enhancers containing H3K4me3 [66]. The co-occupancy of H2A.Z and H3.3 is thought to facilitate chromatin opening to enable gene expression (Figure 2) [65]. This is particularly notable at the +1 nucleosome, where the presence of H2A.Z and H3.3 promotes the unwrapping of nucleosomal DNA [67]. However, the genes where this mechanism acts and whether it is essential for pluripotency or differentiation are unknown.

### 4.4. H2A.Z Promotes RNA pol II Pausing at Promoters of Active Genes

In vitro, it takes longer for RNA pol II to pass through a nucleosome containing H2A.Z compared with one containing canonical H2A [38]. In vivo, H2A.Z occupancy at the +1 and +2 positions correlates with RNA pol II pausing (Figure 2) [39]. In mESCs, H2A.Z plays a direct role in regulating RNA pol II pausing at promoters of active genes [40]. At active genes that are marked by H3K4me3, H2A.Z depletion causes a decrease in RNA pol II pausing and pausing factor NELF at promoters, an increase in elongating RNA pol II in gene bodies, and an increase in gene transcription [40]. Thus, H2A.Z presence at active promoters promotes RNA pol II promoter pausing and acts, essentially, as a repressor of active genes, thereby reducing transcriptional noise. In future, it will be important to examine whether the reduction in H2A.Z protein levels upon differentiation in lineage-specifying genes decreases RNA pol II pausing, thereby increasing the transcription of these genes.

### 4.5. Loss of H2A.Z Alters Chromatin Structure

Differentiation is associated with major changes in nuclear and genomic organization, such as a general decrease in chromatin accessibility and an increase in facultative heterochromatin. For example, during differentiation of human ESCs (hESCs) into human neuroectodermal cells, chromatin becomes globally compacted as differentiation progresses [23]. At the local level, the formation of nucleosome-depleted regions (NDRs) at the TSS of neuroectodermal genes coincides with their increased expression. Interestingly, the TSSs that become NDRs are enriched for H2A.Z in hESCs, which suggests that the loss or destabilization of this H2A.Z-containing nucleosome is likely involved in promoter activation [23].

During the differentiation of mESCs to lineage-committed endoderm/hepatic progenitor cells, H2A.Z-containing nucleosomes are removed preferentially at promoter, exonic, and 5′-untranslated regions of activated genes. This loss depends on the binding of the pioneer transcription factor Foxa2 to nucleosomal DNA wrapped around H2A.Z-containing nucleosomes and is followed by the recruitment of the ATP-dependent remodeling complexes SWI/SNF and INO80 [21]. Conversely, in *C. elegans*, activation of foregut genes requires the deposition of H2A.Z at their promoters, and this process is mediated by the FoxA transcription factor family member PHA-4. This recruitment of H2A.Z also requires the TIP60 acetyltransferase and SRCAP chromatin remodeling complex (see below) [68]. Experiments using in vitro and mouse model systems have shown that H2A.Z-containing nucleosomes block the binding of the intestine-specific transcription factor CDX2 and prevent terminal differentiation into different lineages of intestinal progenitor cells [53].

### 4.6. Different H2A.Z PTMs Are Associated with the Repression or Activation of Differentiation Genes

H2A.Z methylation generally correlates with inactive chromatin and other histone repressive marks (Figure 2) [8]. Upon induction of neuronal differentiation, induced by treatment of mESCs with retinoic acid or depletion of Nanog, H2A.Z monomethylation on lysines 4 and 7 by SETD6 increases significantly. In undifferentiated mESCs, H2A.ZK7me1 is found at the promoters of repressed differentiation marker genes but, upon activation following differentiation, it is removed and replaced by the acetylation mark [69].

During muscle differentiation, expression of *MyoD* requires H2A.Z acetylation. For example, whereas a nonacetylatable mutant of H2A.Z can be incorporated into the *MyoD* promoter, the promoter remains in a chromatin-compacted state and the gene cannot be activated [27]. In *Drosophila*, acetylation of H2A.Z is mediated by TIP60 and is required for Notch target gene activation, which is essential during embryonic and postnatal development [59]. Therefore, H2A.Z acetylation plays an important role in the expression of both pluripotent and differentiation-specific genes (Figure 2).

### 4.7. H2A.Z Regulates the Establishment of Poised Promoters

**Figure 3:**
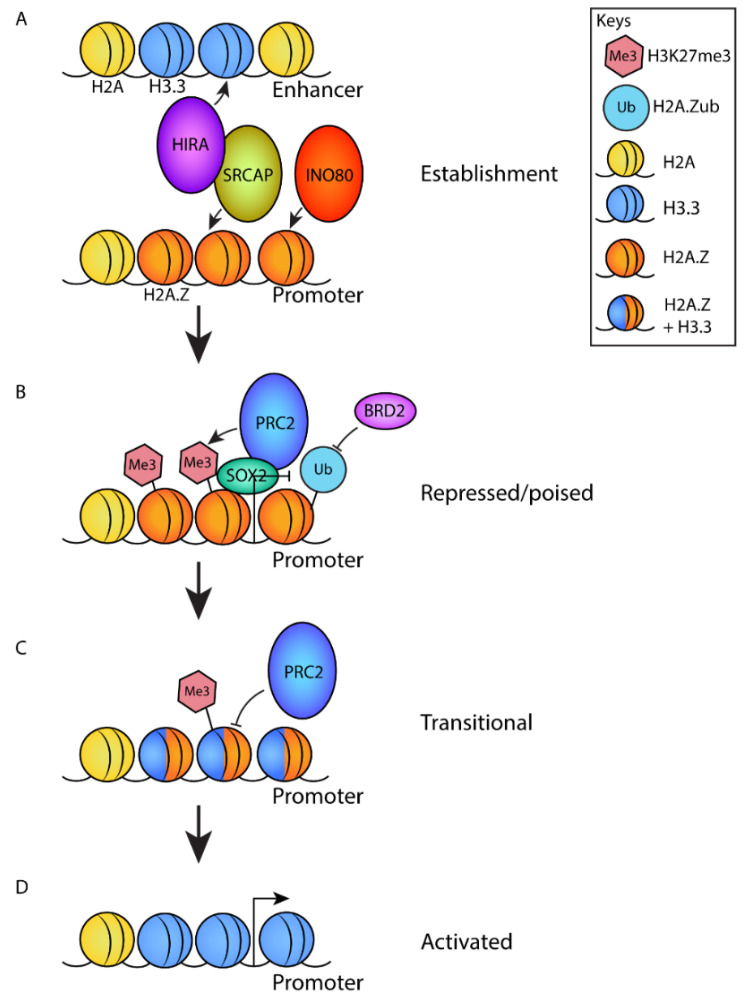
A model describing the role of H2A.Z in the establishment and subsequent activation of poised promoters of differentiation genes. (**A**) At the pluripotent stage, H2A.Z is deposited at promoters by the chromatin remodeling complexes SRCAP and/or INO80. SRCAP associates with the H3.3 deposition complex HIRA, which deposits H3.3 at enhancers to establish a poised state. (**B**) H2A.Z deposition promotes the recruitment of PRC2 through an interaction with SOX2, which promotes the deposition of H3K27me3. H2A.Z is ubiquitylated, which blocks the recruitment of transcriptional activator BRD2. Combined, this yields a repressed/poised chromatin state. (**C**) Upon activation signals that induce differentiation, H3.3 is deposited at promoters. This causes an opening of chromatin structure and represses the activity of PRC2 resulting in the removal of H3K27me3. This results in a ‘transitional’ state. (**D**) Finally, the removal of H2A.Z (by an unknown mechanism) promotes gene activation.

Bivalent domains at poised promoters are established in the blastocyst stage when lineage differentiation begins. Bivalent domains harbor both inactive and active epigenetic signatures, H3K27me3 and H3K4me3, respectively, within the same nucleosome and usually occur at promoters and enhancers [47]. As stated above, bivalent promoters are in a ‘poised’ state, i.e., neither in a permanent ‘on’ nor ‘off’ state. Bivalent domains are thought to primarily control lineage-specifying genes that are transcriptionally activated by the removal of H3K27me3 from the bivalent domain upon differentiation. Thus, bivalency can direct pluripotent cells to specific cell fates. H2A.Z is enriched at bivalent repressed promoters and is essential for the establishment of bivalent domains (Figure 3) [22,51,52,58,70,71,72]. Specifically, the loss of H2A.Z causes impairment in H3K27me3 deposition at bivalent promoters, where H2A.Z is normally located, leading to an increase in gene expression [73]. Polycomb-repressive complex 2 (PRC2) is responsible for mediating H3K27 methylation [47]. H2A.Z is required for the proper localization of the PRC2 subunit SUZ12 [51,73] and the catalytic subunit EZH2 [73]. PRC2 is not required for proper H2A.Z localization [70,73], which suggests that H2A.Z acts upstream of PRC2 when recruiting the complex to chromatin. In addition to recruiting PRC2, incorporation of H2A.Z increases the methylation activity in the PRC2 complex. Interestingly, H2A.Z enhances PRC2 enzymatic activity by facilitating chromatin compaction through its extended acidic patch. This shows that a higher-order compacted chromatin structure is the preferred substrate for PRC2 [70]. Therefore, H2A.Z regulates differentiation by regulating PRC2 recruitment as well as its methylation activity at chromatin (Figure 3).

Because H2A.Z and SUZ12 do not interact directly [51], other proteins are likely to be involved in recruiting PRC2 via H2A.Z. Such a role has been suggested for SOX2, which is a transcription factor involved in promoting self-renewal and neuronal development. SOX2 interacts directly with both H2A.Z and PRC2, thereby bridging H2A.Z and PRC2 and recruiting the complex to specific target sites (Figure 3) [72].

Activation of poised promoters requires the removal of H2A.Z. For example, activation of retinoic acid receptor γ-regulated promoters induces the loss of H2A.Z, which coincides with the removal of SUZ12 and H3K27me3 [22].

At poised promoters, H2A.Z does not act alone. A mechanism has been described, whereby H2A.Z and H3.3 regulate the activity of poised promoters through opposing functions. H2A.Z promotes chromatin compaction and H3.3 opposes this compaction, both in vitro and in cells. In mESCs, retinoic-acid-regulated promoters are repressed by H2A.Z-containing nucleosomes, become transiently primed by the incorporation of H3.3 by forming hybrid nucleosomes following induction by retinoic acid, and finally, become fully activated by the removal of H2A.Z [74]. Further, H2A.Z promotes PRC2 activity and H3K27 methylation, whereas the incorporation of H3.3 into H2A.Z-containing chromatin impairs PCR2 activity by removing the repressive mark to enable gene activation [70]. The HIRA complex, a specific H3.3 chaperone, has been recently shown to interact directly with SRCAP, one of the remodeling complexes that deposits H2A.Z. Through this collaboration between SRCAP and HIRA, hybrid H3.3–H2A.Z nucleosomes can be assembled to prime a promoter for subsequent activation (Figure 3). Notably in mESCs, such priming is important for the activation of genes required for neuronal development [75].

The acidic patch of H2A.Z is essential for its function during development and during the blastocyst stage [13]. The acidic patch has been found to be essential for bivalent gene regulation [70]. Expression of an H2A.Z acidic patch mutant causes a decrease in the incorporation of H2A.Z into the promoter +1 nucleosome and an increase in the expression of bivalent, but not active, H3K4me-marked genes, along with a corresponding decrease in H3K27me3. Interestingly, mutant expression affects the gene expression of only bivalent but not active genes [70]. In one study, the dynamics of chromatin were altered in the H2A.Z acidic patch mutant: the kinetics were faster in the mutant compared to the wild-type H2A.Z, and this change was reflected in a corresponding increase in the amount of H3.3 at promoters. These changes in chromatin dynamics could explain the increase in gene expression observed when the acidic patch mutant of H2A.Z is incorporated into the promoter [76].

H2A.Z PTMs have also been shown to direct bivalency. In particular, H2A.Z monoubiquitylation (H2A.Zub; Figure 2) found at bivalent domains is necessary for polycomb binding and the maintenance of gene repression by preventing the binding of the tandem bromodomain protein BET family member and transcriptional activator BRD2 (Figure 3). BRD2 is required for the activation of developmental genes, including *Brachyury*, *Wnt3a*, *Mesp1*, *Pax6*, and *Sox1* [77]. BRD2 is a direct interactor of H2A.Z nucleosomes, which preferentially binds to nucleosomes containing acetylated H2A.Z in vitro and in cancer cells [64,78].

## 5. Heterochromatin Formation by H2A.Z

While much attention has been given to the role of H2A.Z in the regulation of gene expression during differentiation, H2A.Z is also found at heterochromatic regions, including pericentric heterochromatin (PHC), which flanks centromeres and telomeres [43,79,80,81,82]. PHC is a major component of the nucleus that impacts nuclear and chromosome organization, and its spreading can silence gene expression [83]. Therefore, the proper formation of heterochromatin is a critical component in early development. In the early stages of mouse development, H2A.Z is targeted to PHC where it is involved in recruiting HP1α and promoting the formation of heterochromatin (Figure 1) [43,80,81]. The H2A.Z acidic patch is responsible for this interaction [16,84]. In cell lines, H2A.Z is required for proper chromosome segregation, which relates, in part, to its role in maintaining PHC stability [81]. H2A.Z is also located at centromeres and, in mouse trophoblast stem cells, its abundance at centromeres fluctuates, such that the highest concentration occurs at mitosis when it is required for chromosome segregation [82]. Monoubiquitylation of H2A.Z is also associated with heterochromatin [85].

## 6. Regulation of the Epithelial–Mesenchymal Transition by H2A.Z

The epithelial–mesenchymal transition (EMT) is a profound example of the cell plasticity that is crucial for embryonic development and cancer. In development, the EMT occurs during implantation and embryonic gastrulation, and gives rise to the mesoderm, endoderm, and mobile neural crest cells. By regulating the expression of epithelial and mesenchymal genes, H2A.Z.1 but not H2A.Z.2 (see below) is a master regulator of the EMT in canine and human model cell lines [86]. Specifically, in epithelial cells, H2A.Z at mesenchymal gene promoters keeps them in a repressed state but, upon the transition to the mesenchymal state by exposure to cytokines, H2A.Z is removed from these promoters, enabling their activation [86]. Whether H2A.Z is required for the EMT in vivo is unknown (Figure 1) because H2A.Z-deficient mouse embryos die before gastrulation, when the EMT is critical.

## 7. Different Functions of H2A.Z Isoforms during Development

Differences in H2A.Z function are also conferred by the presence of several H2A.Z variant forms. Although H2A.Z.1, but not H2A.Z.2, is essential for early mouse development, studies have shown that H2A.Z.1 and H2A.Z.2 play different roles following the blastocyst stage. For example, H2A.Z.2, but not H2A.Z.1, has important functions in brain development. H2A.Z.2 is required for the proper development of microglia in the embryonic cerebellum of the mouse, where it is incorporated into the promoter of the chemokine gene *Cxcl14* to inhibit its expression in neural precursor cells. H2A.Z.2 promotes the H3K9me2 modification to repress the expression of this gene [24]. Experiments using zebrafish and mESC-derived melanocyte models have shown that H2A.Z.2, but not H2A.Z.1, has a role in directing neural crest cells to melanocytes. H2A.Z.2 promoter incorporation increases the expression of the key melanocyte determinant gene *mitf* in response to upstream activating signals [29]. The loss of H2A.Z.2, but not H2A.Z.1, in *Xenopus* embryos mimics the human developmental disorder Floating-Harbor syndrome, which causes craniofacial defects. This selectivity appears to be mediated by a higher proportion of H2A.Z.2 versus H2A.Z.1 at enhancer regions that regulate genes involved in facial morphogenesis. Remarkably, this selective role of H2A.Z.2 is caused by a single amino acid residue difference at position 38 (T rather than S) [87].

## 8. Complexes That Regulate Genome Localization of H2A.Z during Development

**Figure 4:**
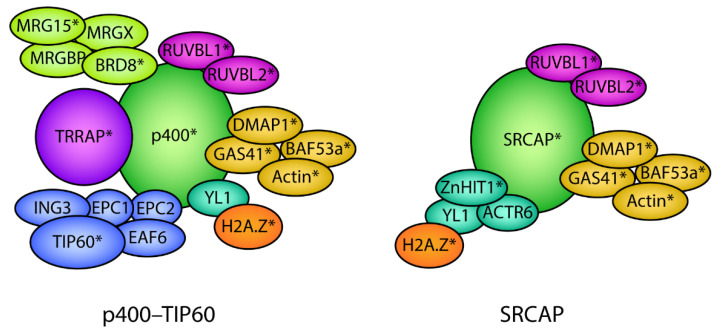
Protein subunit composition of the p400–TIP60 and SRCAP chromatin remodeling complexes. The p400–TIP60 and SRCAP complexes are composed of multiple protein subunits that are assembled into the complex as part of functional modules depicted by the different colors. Several subunits are shared between the p400/SRCAP and SRCAP complexes depicted by the same colors. H2A.Z associates with p400/TIP60 and SRCAP via the shared histone chaperone YL1. * denotes subunits that have been shown to play a direct role in development.

The targeting of H2A.Z to the correct genomic locations at the right time underpins the role of H2A.Z during development. Although it is not well understood how intrinsic and extrinsic signals guide H2A.Z, it is known that directed chromatin incorporation requires ATP-dependent chromatin remodeling complexes. The remodeling complexes that deposit H2A.Z are part of the INO80 SNF2 family of chromatin-remodeling complexes and are called SRCAP and p400–TIP60 in higher eukaryotes. These complexes comprise a number of subunits that are assembled into the complex as part of functional modules, each with unique characteristics that contribute to the overall function of the complex. The composition of the SRCAP and p400–TIP60 complexes is shown in Figure 4.

SRCAP and p400–TIP60 are involved in catalyzing the exchange of H2A with H2A.Z in an ATP-dependent manner. The mechanism involves the replacement of nucleosomal H2A–H2B dimers with chaperone-bound H2A.Z–H2B dimers and occurs in a stepwise manner. The remodeling complex first recognizes the acidic patch of H2A.Z. Next, it replaces one histone dimer to create a heterotypic H2A.Z–H2A nucleosome, followed by the replacement of the second histone dimer to create a homotypic H2A.Z nucleosome [88,89]. ATP hydrolysis is mediated by the SRCAP [89,90] and p400 [91] ATPase subunits. Both SRCAP [92,93,94] and p400 [95] mediate the genome-wide incorporation of H2A.Z. It remains unknown which remodeler is used (and what determines this) in different developmental stages and specific chromatin contexts, but this is likely to be mediated by the unique subunits of each of the different complexes.

### 8.1. The p400–TIP60 Complex Plays Many Roles during Development

The p400–TIP60 complex has been shown to play multiple, essential roles involved in promoting both pluripotency and differentiation during development. The role of the complex has been inferred mainly from the analysis of individual subunits, including studies to determine their expression levels throughout development and the effects of their depletion or mutation. Components in the p400–TIP60 complex (Figure 4) have been found to regulate the differentiation of several tissue types, such as oligodendroglial terminal differentiation (p400) [96], Schwann cell development (p400) [97], neuronal differentiation in *C. elegans* (MRG15) [98,99], differentiation in mouse neural progenitor cells into neural lineages (MRG15) [100], and adipogenesis (BRD8) [101]. p400 is also required for hematopoietic stem cell (HSC) development [102] and for the maintenance of neuroblasts [103]. Mirroring the roles of H2A.Z, the p400–TIP60 complex has been implicated in the promotion of the pluripotency state and repression of developmental genes [104,105,106,107,108,109,110,111,112,113].

Several studies have connected the activity of p400–TIP60 with H2A.Z deposition during development. Mouse terminal oligodendroglial differentiation to myelin is dependent on p400. Depletion of p400 affects the oligodendroglia gene expression network and, in particular, the decrease in the expression of the key transcription factor MYRF. p400 is targeted to the promoter and enhancer regions of *Myrf* by the transcription factor SOX10, and the incorporation of H2A.Z follows p400 binding [96]. In *Drosophila*, knockout of p400, TIP60, or H2A.Z causes the same defects in the Notch pathway during Notch-mediated tissue growth [59], which suggests that these three proteins are functionally connected. TIP60 is an acetyltransferase important for acetylating H2A.Z. For example, TIP60 is essential for the maintenance of mouse HSCs, as it is required for the expression of c-Myc target genes. H2A.Zac is enriched at TIP60 chromatin-bound regions, and depletion of TIP60 causes a decrease in H2A.Zac without an overall change in the occupancy of H2A.Z. This decrease in TIP60-mediated H2A.Z acetylation is responsible for a decrease in c-Myc target gene expression [102].

The roles of p400–TIP60 in development are not always linked with H2A.Z. TIP60 can acetylate a wide range of other proteins, including histone H4 [77] and transcription factors [114,115,116]. Acetylation of the transcription factor SOX4 by TIP60 is essential for promoter activity at the onset of myoblast differentiation [116]. Interestingly, knockout of TIP60 and mutations in its acetyl transferase catalytic domain do not cause the same defects. As examples, the loss of TIP60 catalytic activity results in embryoid body morphology defects because of the impaired induction of mesodermal and endodermal markers, whereas the knockout of TIP60 results in lethality that affects pluripotency and self-renewal [105]. It is possible that TIP60 is required for important protein–protein interactions with interacting partners of the p400–TIP60 complex. p400–TIP60 also regulates gene expression by interacting with key developmental transcription factors, such as c-Myc [117], which coregulates the expression of genes with TIP60 in mESCs [104].

Interestingly, a similar phenomenon is seen for the ATP-hydrolyzing activity of p400. Depletion of the p400 subunit disrupts mESC morphology and expression of pluripotency genes, but loss of p400 ATPase activity does not [105]. Thus, it is likely that the p400-TIP60 complex plays multiple roles during development and that not all of these relate to its H2A.Z-remodeling activity. Further, subcomplexes of p400–TIP60 may be involved in different processes. A recent study compared the knockout of several different components in the p400–TIP60 complex in mESCs. The authors found that the individual knockout of TIP60, EPC1, DMAP1, or p400 (Figure 4) produced different effects by up- or downregulating the expression of different subsets of genes [118]. Thus, the function of p400–TIP60 in depositing H2A.Z during different developmental stages remains unclear.

ANP32e is an H2A.Z-specific histone chaperone that has been suggested to remove H2A.Z from chromatin in cooperation with the p400–TIP60 [119] and INO80 [120] chromatin remodeling complexes. While ANP32e has been shown to remove H2A.Z from chromatin in mouse embryonic fibroblasts (MEFs) [119] and reduce steady-state chromatin levels of H2A.Z in mouse neurons to regulate memory formation [121], ANP32e-knockout mice develop normally [122]. ANP32e deletion in MEFs causes upregulation of differentiation genes [123] and approximately 30% of ANP32e-knockout zebrafish survive to adulthood [42]. Further, in zebrafish embryos, loss of ANP32e causes an increase in H2A.Z incorporation and a concomitant decrease in DNA methylation at non-promoter regions (that contain transcription factor and CTCF binding sites) that would normally acquire DNA methylation in the presence of ANP32e. The increase in H2A.Z at promoters and enhancers results in the precocious expression of developmental regulator genes following zygotic genome activation. ANP32e injection into zebrafish embryos causes a depletion in H2A.Z and a corresponding increase in DNA methylation, largely at genomic regions that are normally developmentally reprogrammed. Therefore, H2A.Z prevents DNA methylation to poise parental genes, either to be activated or repressed following transcriptional onset [42].

### 8.2. SRCAP Plays a Role in Development

Knockout of components in the SRCAP complex has been shown to cause defects in the differentiation of different lineages, including neurons [75], muscle tissue [26], and prenatal heart cells [124]. Mutations in SRCAP cause defects in craniofacial development [87] and are associated with the mental illness bipolar disorder [125]. In contrast to p400–TIP60, SRCAP has no identified molecular roles other than H2A.Z chromatin deposition. Thus, it is assumed that the molecular role of SRCAP in development is solely to facilitate H2A.Z deposition.

In mESCs, SRCAP loss causes a global reduction in H2A.Z occupancy in chromatin [75]. In contrast, the loss of p400 does not induce the same effect, which suggests that SRCAP is the key remodeler responsible for depositing H2A.Z in mESCs [107]. As described above, SRCAP has been shown to associate with the H3.3 deposition complex HIRA to assemble hybrid H3.3–H2A.Z nucleosomes to prime promoters required for neuronal differentiation for subsequent activation (Figure 3) [75].

Truncation mutations in SRCAP cause an inability to localize to the nucleus, which influences neural crest gene expression programs and causes craniofacial defects. As described above, this results from improper H2A.Z.2 chromatin incorporation into AT-rich enhancers [87]. In *Drosophila*, the knockdown of maternal Domino, an SRCAP homolog, also causes a reduction in H2A.Z at TSSs and impairs the expression of housekeeping genes at ZGA. Notably, the deposition of H2A.Z precedes ZGA and, therefore, Domino is essential for the de-novo establishment of ZGA transcriptional programs [41].

The GAS41 subunit is a shared subunit between p400–TIP60 and SRCAP (Figure 4). GAS41 recognizes acetylated H3K27 and H3K14, promoting the recognition of bivalent gene promoters by SRCAP to incorporate H2A.Z and maintain the poised state. Consequently, knockdown of GAS41 results in the derepression of these poised differentiation genes. Notably, defects in H2A.Z chromatin deposition caused by GAS41 depletion are dependent on the SRCAP subunit ZnHIT1, and not p400 [107].

ZnHIT1 is a subunit that is unique to SRCAP (Figure 4) and has been shown to be essential for mediating H2A.Z deposition by SRCAP [31,126]. A key feature of HSCs is that a proportion remains in a dormant cell cycle state, termed quiescence. This is important for sustaining an adequate lifetime supply of blood. ZnHIT1 is essential for maintaining chromatin accessibility of distant enhancers of important HSC-quiescence genes needed for their expression. The knockout of both H2A.Z.1 and H2A.Z.2 mimics the loss of ZnHIT1 and results in exhaustion of this stem cell pool [127]. ZnHIT1 is also required for the deposition of H2A.Z at the promoters required for the expression of key genes involved in oxidative metabolism and mitochondria maturation that are expressed in mouse heart development [124], and for muscle differentiation [26]. Finally, in Lgr5+ intestinal stem cells (ISCs), removal of ZnHIT1 causes a global decrease in H2A.Z abundance, specifically at TSSs, which affects the expression of genes needed for self-renewal and differentiation [31].

SRCAP is required for the self-renewal and maintenance of mouse ISCs. Intriguingly, SRCAP recruits the transcriptional regulator REST to the *Prdm16* promoter to induce the expression of a peroxisome proliferator-activated receptor, a transcription factor required for preserving ISC stemness. Whether H2A.Z participates in this activation process is unknown [128]. SRCAP [124] or p400–TIP60 [129] may also regulate the expression of the H2A.Z gene. Therefore, future studies should confirm whether the loss of H2A.Z from chromatin in SRCAP or p400–TIP60 knockouts is a direct or an indirect effect.

### 8.3. Other Remodeling Complexes Can Target H2A.Z

INO80 is the third member of the INO80 SNF2 family of the chromatin-remodeling complex, which includes SRCAP and p400–TIP60. In budding yeast, INO80 has been shown to specifically remove H2A.Z from chromatin [130], but such a role in metazoans has not been clearly established. In naïve mESCs, the binding of INO80 pre-marks gene promoters that become bivalent with H3K4me3 and H3K27me3 upon transition into the primed state. In contrast to yeast, in the primed mESCs state, INO80 recruits H2A.Z, a step that is necessary for the establishment of the bivalent state (Figure 3) [73]. In support of these findings, INO80 is required for the recruitment of H2A.Z and PRC2 to mediate H3K27me3 in poised somatic genes, which maintains their repression in mouse meiotic spermatocytes [131]. The Brg1-associated factor esBAF1 is an ESC-specific ATP-dependent nucleosome-remodeling complex required for the activation of transcription that is needed for mESC renewal and pluripotency. Interestingly, loss of esBAF1 results in the conversion of nucleosomes to subnucleosomal particles and the loss of H2A.Z at previous sites of H2A.Z occupancy. This suggests that either esBAF1 promotes H2A.Z deposition, possibly by facilitating the function of SRCAP or p400–TIP60, or stabilizes H2A.Z-containing nucleosomes [132].

## 9. Conclusions

Histone variants have played a major role in the evolution of complex multicellular organisms. They have expanded the functions of chromatin to facilitate the regulation of gene expression in a precise spatial- and temporal-specific manner, which underpins cell fate decisions. H2A.Z has a particularly important role and, moreover, it is needed throughout pre- and postimplantation development in mammals when key differentiation decisions are made. This shows that the unique structural and functional properties that H2A.Z imparts to chromatin are utilized in many different ways to enable the development of a multicellular organism. It is also intriguing that different metazoans employ H2A.Z differently, indicating that for each species, a unique set of developmental problems need to be solved. It can be postulated that the ‘tool kit’ that H2A.Z provides, through altering chromatin structure and delivering numerous different protein–protein interaction faces by PTMs and the crosstalk with other histone PTMs and histone variants in the same nucleosome, enabled such developmental problems to be solved during evolution. However, many unanswered questions remain, including: What determines whether a gene is positively or negatively regulated by H2A.Z? What are the signaling pathways that direct SRCAP or p400–TIP60 to specific enhancers or promoters to deposit H2A.Z and how does this happen? Which chromatin remodeling complexes remove H2A.Z from chromatin? Notably, although p400–TIP60 has been shown to directly deposit H2A.Z into chromatin in vitro, there is limited evidence that it performs this function in vivo during development. Why do mouse embryos deficient in H2A.Z die around implantation? Is it related to impaired gene regulation or due to chromosome segregation defects? Because H2A.Z-knockout mouse embryos die early, what other important functions does H2A.Z perform during the later stages of development? We look forward to these and other important questions being addressed in the future.
